# Establishing environmental causes of childhood cancer: a view from the IARC Monographs

**DOI:** 10.14324/111.444/ucloe.3600

**Published:** 2026-10-01

**Authors:** Elisa Pasqual, Andrew Kunzmann, Julia Heck, Margaret R. Karagas, Mary K. Schubauer-Berigan

**Affiliations:** 1 International Agency for Research on Cancer (IARC), Lyon, France; 2 College of Health and Public Service, University of North Texas, Denton, TX, USA; 3 Geisel School of Medicine, Dartmouth College, Hanover, NH, USA

**Keywords:** environmental causes, epidemiology, childhood cancer

## Abstract

In 2025, the IARC Monographs programme identified two new modifiable potential causes of childhood acute lymphoblastic leukaemia, with *limited* evidence: automotive gasoline and human cytomegalovirus. Previously, for childhood acute lymphoblastic leukaemia only one potential cause was identified (parental tobacco smoking, with *limited* evidence). The Monographs programme, throughout its history, has identified few other causes for specific childhood cancers. Here, we aim to: i) summarise IARC Monographs classifications relevant for childhood cancer aetiology; ii) describe the evidence that contributed to the recent classifications of automotive gasoline and human cytomegalovirus; and iii) list agents with evidence for an association with childhood cancer that are currently prioritised for an IARC Monographs evaluation in the 2025–2029 period. Several factors have been increasingly identified as potential modifiable causes of childhood cancers. Methodologically, studies have been improved by including assessment of the exposure prior to diagnosis (e.g., use of geocoding methodology or stored neonatal bloodspots) that were instrumental in the automotive gasoline and human cytomegalovirus classifications. Several agents currently prioritised for IARC Monographs evaluation, mainly individual pesticides or air pollution components, present sporadic positive findings for specific childhood cancer subtypes. IARC Monographs evaluation of cannabis smoking is planned for November 2026. Sporadic positive associations for specific childhood cancer subtypes were also reported for cannabis smoking (through parental habits) in case–control studies in the 1990s and early 2000s. Studies informative for cancer hazard identification are likely those able to well define the exposure (e.g., individual pesticides), the window of exposures (e.g., in utero) and the outcome (e.g., avoiding combining childhood cancers).

## Background

Childhood cancer is rare, yet it remains a leading cause of death in children, despite significant improvement in childhood cancer survival rates [1]. It is also an important public health burden, because childhood cancer survivors are at higher risk of chronic diseases, second primary cancers or financial distress [2].

The most common cancer types in children (<15 years old) are leukaemias, with acute lymphoblastic leukaemia (ALL) being the most common paediatric malignancy, Hodgkin lymphoma (HL) and non-Hodgkin lymphoma (NHL) and brain tumours, particularly gliomas and embryonal tumours (e.g., medulloblastomas). There are also other cancer types that tend to occur almost exclusively in children, such as Wilms tumour and other embryonal tumours [1,3].

The incidence of childhood cancer is slowly and steady increasing worldwide [1]. Such trends cannot be entirely explained by advancements in diagnosis, genetics or physiological factors (e.g., parental age), and it is likely that environmental factors, which change over time, play a role [4].

Children can be especially vulnerable to environmental pollutants or biological agents owing to their potentially high exposure (e.g., hand–mouth habits), their developing physiology and longer lifespan to develop long-term health effects [5]. Parental exposure is also a concern in children, owing to potential effects in the pre-conception or pregnancy window [6]. New suspected modifiable causes of childhood cancers are increasingly being reported because of the progress made in the last decade to develop new infrastructure for studying childhood cancer risk factors, including the creation of large consortia and data platforms [7]. However, few environmental or lifestyle causes of childhood cancer have been established or recognised by authoritative public health organisations. Environmental risk factors have been generally downplayed or are less well known compared to genetic factors. For example, a World Health Organization fact sheet on childhood cancer from February 2025 states that ‘very few cancers in children are caused by environmental or lifestyle factors’ [8].

The IARC Monographs programme is recognised worldwide for cancer hazard identification and evaluates the carcinogenicity of ‘agents’, broadly defined as any modifiable factors, as opposed to genetic or pathophysiological factors (e.g., endogenous hormones, diseases) [9]. In 2025, two IARC Monographs Working Groups classified two agents with *limited* evidence for childhood ALL: automotive gasoline [10] and human cytomegalovirus (HCMV) [11]. Before these classifications only parental tobacco smoking was identified as a potential cause for childhood ALL (i.e., with *limited* evidence).

In this commentary we aim to: i) summarise these and previous IARC Monographs evaluations having *sufficient* or *limited* classifications for relevant childhood cancer sites; ii) describe the types of evidence that contributed to the recent automotive gasoline and HCMV classifications, reflecting on research advancement and gaps; and iii) provide the list of agents currently prioritised for IARC Monographs evaluation (2025–2029) that may be relevant to childhood cancer environmental aetiology.

Overall, we aim to stimulate research in this area and raise awareness of known or suspected modifiable environmental causes of childhood cancers.

## How the IARC Monographs evaluations are conducted

Since 1971, the IARC Monographs have identified 135 agents in Group 1 (‘carcinogenic to humans’), 98 in Group 2A (‘probably carcinogenic to humans’) and 329 in Group 2B (‘possibly carcinogenic to humans’). The IARC Monographs classifications follow procedures detailed in the Preamble, which was last revised in 2019 [9,12]. Briefly, three streams of evidence are synthesised and then integrated: evidence from studies with cancer endpoints in humans; evidence from animal studies with cancer endpoints; and mechanistic evidence, which includes evidence that the agent exhibits key characteristics of carcinogens [9].

For each cancer organ or tissue, the evidence from studies with cancer endpoints in humans can be *sufficient*, when a causal association has been established (i.e., a positive association is observed in the body of evidence and chance, bias and confounding can be ruled out with reasonable confidence); *limited* (when there is a credible positive association, but chance, bias and/or confounding cannot be ruled out with reasonable confidence); and *inadequate* (when there is lack of data, studies are of insufficient quality or statistical precision or results are inconsistent, null or negative and there is no explanation for such inconsistency) [9]. A *sufficient* determination in human cancer studies can lead to a Group 1 classification. A Group 1 classification can also be reached when combining *sufficient* evidence in animals and *strong* evidence from human mechanistic studies [9].

## Review of the causes of childhood cancer identified by the IARC Monographs

We extracted the list of agents classified as causes (with *limited* or *sufficient* evidence) for childhood cancers. The IARC Monographs maintain the list of classified agents continuously updated and freely accessible on its website (https://monographs.iarc.who.int/agents-classified-by-the-iarc/), including a list by cancer types for which *sufficient* or *limited* evidence is found.

### Childhood cancer sites with an evaluation

Throughout the history of the Monographs, very few agents have been found with either *sufficient* or *limited* evidence for childhood cancers. Causes of specified childhood cancer have been established, with *sufficient* or *limited* evidence, only for childhood acute myeloid leukaemia (AML), ALL, childhood leukaemia overall and hepatoblastoma (see Fig. 1). Although the evaluations were not restricted to childhood, several agents were identified as causes (with *limited* or s*ufficient* evidence) of cancers common in children (e.g., brain, HL, NHL, Burkit lymphoma) (Fig. 1). Although some causes of ovarian or testicular cancer have been identified, none was specific for germ cell tumours or even more specific for yolk sac tumours or teratomas, which are the subtypes of germ cell tumours more common in children [3].

**Figure 1:**
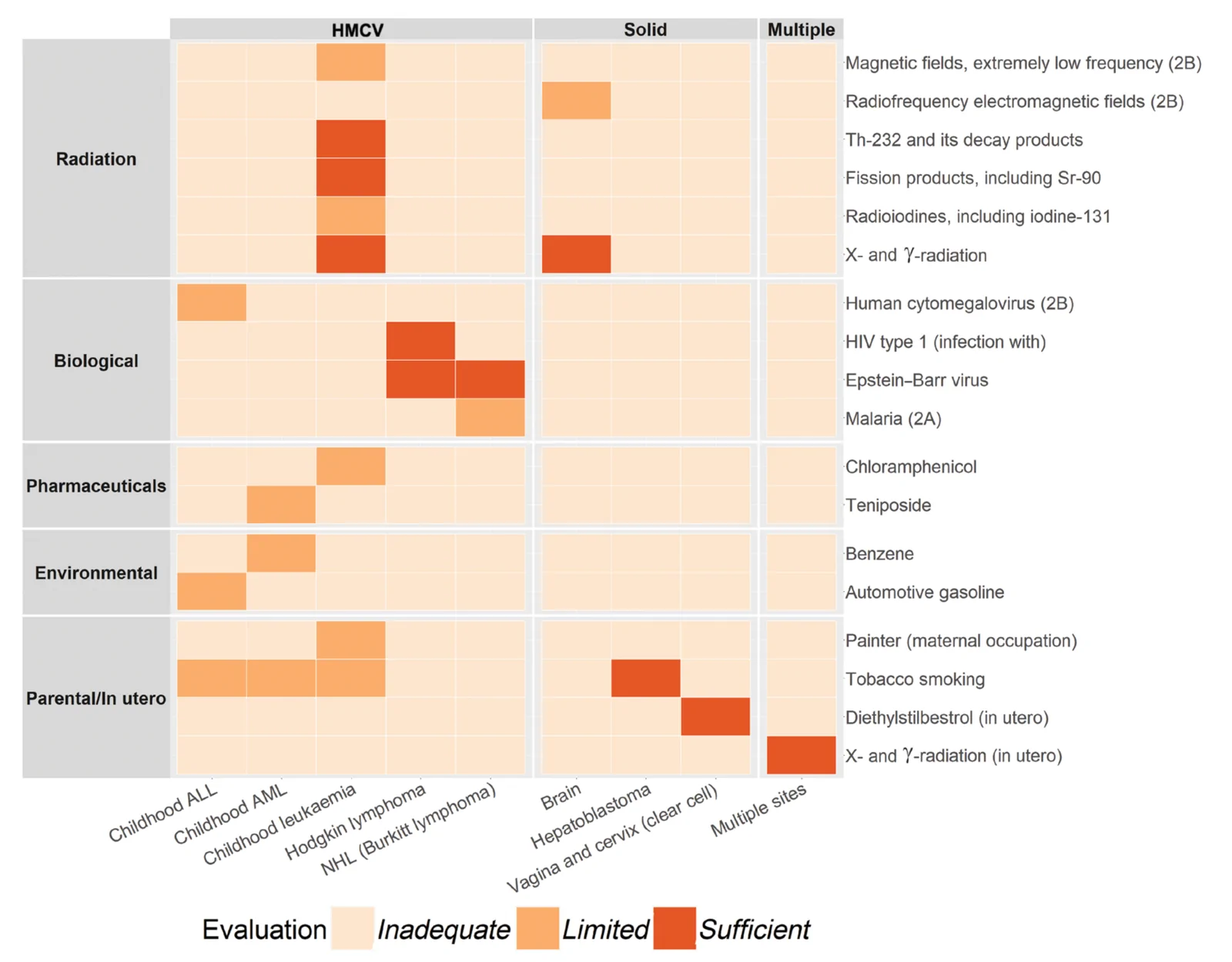
Agents identified with having *limited* or *sufficient* evidence for childhood specific cancer or cancer sites common in children. Agents other than Group 1 have their group classification identified in parenthesis (2A, 2B). Other causes of NHL or lymphoma have been identified by IARC Monographs, but in this figure, we are selecting those that cause NHL subtypes common in children. This figure was generated using data from the IARC online classification, continuously updated and freely accessible from the IARC Monographs websites: https://monographs.iarc.who.int/agents-classified-by-the-iarc/.

### Identified causes of childhood cancer

#### Agents with *sufficient* evidence

*Sufficient* evidence for childhood cancers or for cancer subtypes common in children (such as Burkitt lymphoma, HL, brain tumours) has been determined only for physical (X- and gamma- radiation, fission products) [13] or viral agents [Epstein–Barr virus, human immunodeficiency virus (HIV)] [14] (Fig. 1). Among other agent types, there was *sufficient* evidence that diethylstilbestrol causes clear cell carcinoma of the vagina and the cervix following in utero exposure [15]. Indeed, observing these very rare tumours at young ages in daughters of mothers who used diethylstilbestrol during pregnancy contributed to the strength-of-evidence determination. Although embryonal tumours are common cancers in children, only parental tobacco smoking has been identified as a cause of hepatoblastoma (with *sufficient* evidence) [16]. No agent has been established as causal in other embryonal tumours.

#### Agents with limited evidence

For childhood leukaemia overall, agents listed as having *limited* evidence included parental tobacco smoking [16], chloramphenicol [17], radioiodines (including I^131^) [13], maternal occupational exposure as a painter [18] and extremely low frequency magnetic fields [19] (Fig. 1). There is also *limited* evidence for benzene [20], parental tobacco smoking [16] and teniposide (a chemotherapeutic drug) [21] as causes of childhood AML (Fig. 1). Prior to 2025, only parental tobacco smoking had limited evidence as a cause of childhood ALL [16]. Automotive gasoline and HCMV were each classified in 2025 with *limited* evidence for childhood ALL [10,11].

### Automotive gasoline and HCMV evaluations

#### Overview of the childhood ALL literature for automotive gasoline

Gasoline was classified as carcinogenic to humans (Group 1), based on *sufficient* evidence for adult AML and bladder cancer [10]. The 2025 childhood ALL *limited* classification was based mainly on case–control studies in which automotive gasoline exposure reported by parents (during pre-conception, prenatal or during childhood) was associated with an increased incidence of childhood ALL. In these studies, however, recall bias and chance could not be ruled out [10]. The evidence also included studies using residential address at birth or at diagnosis to estimate distances from gasoline stations, a measure that is not affected by recall bias. These studies indicated an increased incidence of leukaemia in children living within 150 m of service stations, but these studies mainly were on childhood leukaemia overall rather than on childhood ALL specifically.

#### Overview of the childhood ALL literature for HCMV

HCMV was classified as possibly carcinogenic to humans (Group 2B), based on *limited* evidence for childhood ALL [11]. This evidence was based on case–control studies with available pre-diagnostic samples. These were stored neonatal dried blood spots from which HCMV proteins or nucleic acids could be measured [22] or stored maternal, gestational blood samples from which HCMV immunoglobulins could be assessed [23]. Case–control studies measuring HCMV positivity in neonatal stored blood samples used for routine newborn screening (e.g., dried blood spots) were particularly informative, because they ensured the availability of pre-diagnostic data. However, HCMV prevalence is low in neonates, and this affected the statistical precision of the results. Additionally, there were no data to rule out unmeasured confounders potentially related to HCMV maternal or neonatal prevalence in specific populations.

#### Considerations on exposure assessment methods

The IARC Monographs evaluation, following the 2019 revision of the Preamble [9], has moved towards a systematic and detailed review of the exposure assessment in human studies considered in the evaluation. This has resulted in the development of a specific workflow, which was followed for the evaluation of automotive gasoline and HCMV [24]. The novelty of these two recent evaluations, with respect to past childhood cancer evaluations, was the availability of studies with pre-diagnostic measurements, which complemented the evidence from traditional interview-based case–control studies.

Given the challenge of obtaining pre-diagnostic samples in childhood cancer studies, studies traditionally have relied on parental reporting on past exposure, which may be subject to recall bias [25]. Extensive effort has gone into assessing in utero and childhood exposure at different time points in maternal–child cohort studies, but these tend to be inefficient for rare childhood cancer subtypes. Despite ongoing efforts to pool birth cohorts to study childhood cancer outcomes, such as the International Childhood Cancer Cohort Consortium, the statistical power in cohort studies remains low, especially for rarer childhood cancer subtypes [26].

Geographic information systems (GIS)-based studies to assess residential environmental exposure are increasing, and many of the studies of agents currently prioritised for evaluation used in the Monographs also present this type of design (see below and see Table 1). Stored neonatal blood spots are increasingly used to assess biomarkers of environmental exposure [63], such as nicotine, trace elements and persistent organic pollutants [64]. It is important to note, however, that biological-based measurements, especially if taken at a single time point, may still be subject to non-differential exposure misclassification, particularly for agents with short half-lives [25]. Likewise, GIS-based studies might also be affected by exposure misclassification because full residential history is often lacking. Additional non-differential exposure misclassification in GIS-based study could come from modelling uncertainty. It is also important to consider that recall bias might not be a concern for all interview-based studies in case–control studies. For example, reporting of the occupational use of pesticides has not been found to be differential between cases and controls [65]. In the automotive gasoline evaluation, interviewed-based study were not discarded, rather they were evaluated together with residential-based studies [10].

**Table 1.** Environmental agents* prioritised for evaluation by the IARC Monographs programme (2025–2029) with evidence of childhood cancers

Agent (priority); previous IARC classification	Exposures	Childhood cancer
Parental habits and occupations		
Parental tobacco smoking (high); IARC Group 1	Widespread globally	The advisory group noted that new studies that link parental tobacco smoking and childhood cancers have been available since the latest tobacco smoking evaluation [16]. Several recent meta-analyses have highlighted increased risk for maternal smoking and childhood brain tumours [27] or for paternal smoking and childhood ALL [28]. The 1.2 or 1.3-fold risk for pre- and postnatal paternal smoking observed for childhood AML in the pooled analysis of Childhood Leukaemia International Consortium was also highlighted; however, maternal smoking showed associations only in specific ethnic subgroups [29]. In addition, prenatal maternal and paternal smoking were associated with increased risk of neuroblastoma in offspring in a recent meta-analysis [30].
Cannabis smoking; never evaluated	Through exposure to maternal or parental cannabis smoking	The advisory group mainly highlighted positive associations for testicular germ cell tumours, which are the most common cancers in males 15–35 years of age. As regards childhood cancers, although not specifically cited in the Advisory Group report, reviews, including the California state evaluations [31,32], have highlighted that sporadic positive findings were reported in several case–control studies conducted in the 1990s and early 2000s, mostly by the Children’s Cancer Study Group for: - Childhood acute non-lymphoblastic leukaemia - Childhood astrocytoma - Neuroblastoma.
Textile manufacturing industry work; Group 2B	Maternal or paternal occupation	In addition to the adult cancer studies highlighted in the IARC Advisory Group report, several studies were identified that explored the associations between parental occupation and childhood cancer in offspring, using either census data or parental interviews. Associations were reported for parental (maternal and/or paternal) textile manufacturing industry work and childhood central nervous system (CNS) tumours [33–35], paternal or maternal contact with textile dust and Ewing sarcoma [36] or childhood cancer tumours other than leukaemia or CNS tumours [37], maternal textile work and childhood acute non-lymphoblastic leukaemia [38] or childhood soft tissue sarcoma [39].
Outdoor air pollution and its components		
Outdoor air pollution (high); IARC Group 1	Widespread, especially low- and middle-income countries (LMIC)	The IARC Advisory Group reported the available evidence for childhood leukaemia (including subtypes) citing a recent meta-analysis which identified several studies reporting associations between proxies of outdoor air pollution (e.g., traffic density, benzene) and childhood leukaemia, including subtypes (ALL, AML). A stronger risk was found for benzene and AML (RR = 1.84), and other measurements of air pollution were weakly or imprecisely associated [40]. For other childhood cancer subtypes (childhood NHL, childhood CNS tumours, neuroblastoma, other subtypes of childhood cancer), several studies with positive results for air pollution metrics, many also based on geocoding data, are available and identified in a recent scoping review [41].
Ultrafine particles (UFP) (high); never evaluated	Component of air pollution	Only a single study that associated UFP and childhood cancer risk was identified by the advisory group. A case–control study in Canada reported an association between UFP exposure in the first trimester, based on residential air pollution exposure modelling, and overall childhood cancer incidence; however, associations weakened when studying specific subtypes [42].
Acetaldehyde (high); acetaldehyde: Group 2B, acetaldehyde associated with consumption of alcoholic beverages: Group 1	Component of air pollution	The advisory group highlighted the sporadic positive associations between early life exposure to acetaldehyde and several childhood cancers. In a single case–control study of retinoblastoma (<6 years of age) in California, acetaldehyde exposure in the first year of life was found to be associated with increased retinoblastoma risk [43]. Based on residential-based modelling exposure, acetaldehyde in air pollution during the third trimester was associated with Wilms tumour in a single case–control study [44]. The occurrence of the CNS primitive neuroectodermal tumour was also associated with prenatal acetaldehyde exposure in a California case–control study [45]. No additional studies were identified.
Formaldehyde (high); Group 1	Component of air pollution	In addition to the adult studies highlighted by the AG, formaldehyde found in air pollution during pregnancy, as based on residential-based exposure modelling in a single case–control study, has been positively associated with risk of Wilms tumours in offspring (in younger than 6 years of age) [44]. Small imprecise increased associations were found for childhood leukaemia in a similar case–control study in California [46].
NO_2_ (high); never evaluated	Component of air pollution	No specific study on childhood cancer was highlighted by the Advisory Group on childhood cancer. However, a recent meta-analysis identified nine studies associating NO_2_ and childhood leukaemia, including subtypes, with some evidence of increased risk in the dose–response analysis for the highest category of exposure, although this was imprecise [40]. Residential NO_2_ levels were associated with childhood AML in a subsequent study [47]. Few studies explored prenatal windows of exposures, with one study reporting associations for first trimester NO_2_ exposure and childhood ALL, but not for other windows of exposure [48].
Xylene (medium); Group 3	Component of air pollution	The Advisory Group report that findings for human cancer are only sporadic. Regarding childhood cancers, in a single case–control study in California in children (<6 years of age), meta/para-xylene exposure was associated with all germ cell tumours and yolk sac tumours (<6 years of age), especially for exposure that happened in the second trimester. Orto-xylene exposure in second and third trimester was also associated with increased risk of yolk sac tumour [49]. In a similar case–control study in California focused on retinoblastoma (<6 years of age), average meta/para-xylene exposure in utero was associated with increased risk of retinoblastoma [43]. No other study was identified.
Selenium (medium) Group 3	Component of air pollution, diet intake	The Advisory Group report highlighted mixed results for human cancer evidence and reported the challenge of the interpretation of the data in human cancer studies. Childhood cancer studies were not highlighted in the report. For childhood cancer studies, selenium, as a component of air pollution, was analysed in some of the air pollution modelling studies identified for other air pollution components. Some imprecise positive associations were reported for childhood leukaemia. There are also some positive findings for childhood leukaemia in case–control studies with blood measurements of metals, although this is not consistent across all forms of selenium and measurements are taken after diagnosis, raising concern over reverse causation [50].
Carbon tetrachloride (high); Group 2B	Solvents, agricultural fumigants, industrial processes (use now restricted), air pollution	The Advisory Group report cited a single Californian case–control study of neuroblastoma (<6 years of age), carbon tetrachloride exposure in utero (2.5 km buffer) was associated with neuroblastoma risk with a very imprecise 7-fold increase [51]. In addition, parental occupation exposure to carbon tetrachloride, particularly paternal pre-conception exposure, was found to be associated with increased childhood leukaemia and NHL in offspring in the north of England [52]. Of note, the Advisory Group report has highlighted positive and consistent findings for adult NHL.
Physical agents		
Radon-222 and its decay products (high); Group 1	Naturally occurring gas. Exposure occurs at residential levels, especially in areas with high concentrations or in indoor subterranean areas.	The Advisory Group report highlighted the availability of an increased number of studies with respect to radon evaluation in 2009, on childhood leukaemia [13]. These include several recent meta-analyses with positive results, which merit further evaluation [53].
Pesticides		
Pyrethrin and pyrethroid insecticides (high); Group 3 Note: Several other pyrethrins and pyrethroids have been recommended by the Advisory Group to be evaluated and the recommendation is evaluating single pyrethroid and pyrethrins in a single volume, not the class combined.	Insecticide class Use: Worldwide agricultural, veterinary, domestic and public health use (disinfection of buildings, treatment of mosquito nets). Childhood exposure could occur through environment (residence near crop fields), contact with family members occupationally exposed, exposure in residential setting (used in home or gardens).	Few available studies were reported by the Advisory Group. Residences near where pyrethroids were sprayed (600 m buffer) was not associated with childhood leukaemia [54]; however, permethrin (see below) was associated. In single studies, parental-reported use of insecticidal shampoo containing pyrethroids was associated with acute childhood leukaemia (ALL and ANLL) [55] and urinary levels (at diagnosis) of pyrethroids insecticides were higher in cases of childhood ALL [56].
Permethrin (high); Group 3	Class: pyrethroid pesticide (see above) .Childhood exposure also could occur through the application of shampoo for treatment of head lice and scabies.	Few available studies were reported by the Advisory Group. Increased risk was found in the geocoding-based childhood leunkemia study based in California [54], but not in the Californian childhood ALL study using measures of permethrin in household dust [57]. Parental-reported exposure to permethrin was associated with childhood ALL and AML in Brazil [58].
Chlorpyrifos (high); never evaluated	Organophosphate insecticide still registers in the US but registration was cancelled in the European Union since 2020. Exposure in children occurs through contaminated water and food.	No specific childhood cancer study was highlighted by the Advisory Group. In the Californian geocode state-wide case–control study chlorpyrifos was associated with childhood ALL (<6 years of age) in a single pesticide-adjusted model [59]. No other study was identified.
Malathion (high); Group 2A	Broad-spectrum organophosphate insecticide, the most used in the US, is also used for mosquito control. Exposure in the general population occurs from food, the environment or from personal use.	In the Californian geocode state-wide case–control study malathion was associated with childhood ALL (<6 years of age) only in the single pesticide-adjusted model, association weakened in the fully adjusted model [59]. No other study was identified.
Pendimethalin (medium priority); never evaluated	Class: herbicide Use: crop and non-crop uses (e.g., urban residential environments). Childhood exposure could occur through the environment (residence near crop fields), contact with family members who are occupationally exposed, but also secondarily due to the ingestion of contaminated fruit and vegetables.	Two studies on childhood leukaemia were cited by the Advisory Group report. A suggestive elevation was reported in the Californian geocode nationwide case–control study (childhood leukaemia <16 years of age) [54]. Pendimethalin in household dust was not associated with childhood ALL [57]. In addition, in the Californian geocode state-wide case–control study malathion was associated with childhood ALL (<6 years of age) only in the single pesticide-adjusted model, association weakened in the fully adjusted model [59].
Biologicals		
*T. gondii* (medium); never evaluated	Parasite responsible for toxoplasmosis. It can be transmitted during pregnancy to the fetus, and it is estimated that the prevalence of toxoplasmosis is around 30% in pregnant women worldwide.	Childhood brain tumour Childhood leukaemia Few available studies were found by the Advisory Group. Studies, mostly from LMIC, have identified higher *T. gondii* immunoglobulin G seroprevalence in cases of childhood brain tumours or leukaemia compared to controls [60,61].
Human papillomavirus (HPV) (high); HPV type 16, 18, 31, 33, 35, 39, 45, 51, 52, 56, 58, 59 are Group 1 Note: HPV was not nominated, but the Advisory Group recommended systematic re-evaluation of Group 1 agents	Children could be infected through vertical transmission, including perinatally during delivery through contact with an infected maternal tract, non-sexual horizontal and sexual abuse transmissions [62].	Retinoblastoma Mechanistic evidence could support a role of HPV in retinoblastoma onset through HPV binding of p53 and retinoblastoma tumour-suppressor proteins. Conflicting results exist regarding the testing of the presence of the virus in retinoblastoma samples and some studies have linked maternal HPV infection to retinoblastoma development [62]

*Environmental agents are defined as modifiable factors to which children are exposed in the environment. The exposure could also occur through parental environmental exposure.

The references listed in this table are not meant to be comprehensive, and a full Monographs evaluation, which includes a systematic literature review, is needed to establish whether the factors listed are causal or not.

#### Considerations on window of exposure

The use of neonatal blood spot samples has also been instrumental in providing molecular evidence that a proportion of childhood ALL and AML originates in utero. Indeed, evidence of pre-leukaemic clones has been detected in stored neonatal blood spots of children who later developed ALL and AML, although the detection of pre-leukaemic clones has varied across ALL and AML subtypes [66]. The two-hit hypothesis, which was first developed in 1971 by Knudson to explain heritable and non-heritable retinoblastomas, considers that cancer is caused by two mutational events on both alleles of a tumour suppressor gene [67]. The two-hit hypothesis applied to childhood leukaemia development considers that childhood leukaemia originates in utero with the development of pre-leukaemic clones and progresses postnatally into overt leukaemia after a second-hit mutation. Therefore, most recent childhood cancer research has focused on testing associations by distinct windows of exposure (e.g., prenatally and postnatally) [6]. This was the case also for the evidence available for HCMV, based on samples related to prenatal exposure (maternal HCMV positivity during pregnancy) or perinatally (positivity of dried blood spot samples). The interview-based childhood ALL studies available for gasoline also assessed these different windows of exposure. However, the identification of risk factors acting as exposures during pregnancy is challenged by the effect of live birth bias, which has been found to be operating predominantly downwards [68]. In addition, exploring associations across different critical exposure windows (pre-conception, in utero, postnatal) for parental occupation or residential exposure is challenging in practice because parents do not often change jobs or residence during a pregnancy.

#### Confounding and bias assessment considerations

The Working Group concluded that bias, confounding or chance could not be excluded with reasonable confidence when evaluating the carcinogenicity of automotive gasoline and HCMV on childhood ALL. For automotive gasoline, recall bias and chances could not be fully excluded, as there were few GIS-based studies focused on childhood ALL [10]. For HCMV, there were concerns about chances and residual confounding [11]. Indeed, disparities in childhood cancer incidence by socioeconomic status and race or ethnicity are well documented and reasons for such disparities are not fully explained [5,69]. Further research investigating racial and ethnicities disparities in childhood cancer incidence could clarify the aetiological role of specific environmental exposures. Recall bias, imprecision of the estimates and residual confounding are common issues in interpreting childhood cancer studies. In addition to those, downward biases could exist from non-differential exposure misclassification and live birth bias, as mentioned previously. In evaluating the studies, the IARC Monographs Working Group assessed the impact of these biases. The IARC Monographs have also provided guidance on analysing biases in cohort and case–control studies [25]. Evidence synthesis efforts, including meta-analysis, could adopt some of these tools to further strengthen their results based on these types of considerations.

## Review of the evidence for childhood cancer for environmental agents identified with high or medium priority for IARC Monographs evaluations (2025–2029)

### How agents are prioritised to be reviewed by the IARC Monographs

In 2024, an IARC Advisory Group met to prioritise the agents to be reviewed by the IARC Monographs in the 2025–2029 five-year cycle [53,70]. Agents were nominated for review by the general public and the broader public health and scientific community. The reviews of nominated agents were carried out by experts in cancer hazard identification from various countries, who were asked to identify major publications and data that could lead to a new or revised IARC Monographs classification. For this assessment, contrary to a Monographs evaluation, the review of the literature is not systematic. The Advisory Group identify key publications based on their expertise in the field, on the evidence highlighted in the nomination forms or on literature searches. Their assessment is written in a report that it is publicly available [53]. For this review, we identified publications mainly through this report.

On the list of agents prioritised for evaluation by the IARC Monographs in 2025–2029, several appear to have studies in relation to childhood cancer risk [70], including several environmental agents such as outdoor air pollution and its components, some pesticides, biological agents and parental tobacco smoking (see Table 1). The references listed in Table 1 are not meant to be comprehensive, and a Monographs evaluation is needed to establish whether the factors listed are causal or not. However, the list of agents reported are consistent with agents that have been highlighted in other recent reviews [41,71,72]. Of note, some of these environmental factors can also impact childhood cancer clinical outcomes (e.g., survivorship) [5].

### Group 1 agents prioritised for re-evaluation

Tobacco smoking, outdoor air pollution and radon-222 and its decay products (hereafter shortened to radon-222) were nominated and recommended for re-evaluation by the IARC Monographs programme with a high priority. The rationale for revaluation is to determine whether new cancer sites should be classified as having *sufficient* or *limited* evidence of human carcinogenicity. Tobacco smoking, outdoor air pollution and radon-222 present evidence for some childhood cancer subtypes (Table 1). Given that all nominated Group 1 agents presented evidence for additional cancer sites, the Advisory Group recommended a systematic re-evaluation of all Group 1 agents.

Indeed, evidence has grown for parental tobacco smoking (currently *sufficient* for hepatoblastoma and *limited* for childhood leukaemia overall) [16] and subsequent risk of childhood brain tumours, neuroblastoma, childhood ALL or AML. The advisory group pointed out several meta-analysis or pooled analysis that report evidence of dose–response relationships for paternal or maternal smoking (Table 1) [70]. Dose–response relationships are particularly challenging to observe in childhood cancer studies as they could be affected by live birth bias. Indeed, exposure to toxicants during pregnancy, especially at high doses, could result in spontaneous abortion, causing a downward bias [68]. To the best of our knowledge, only one study used tobacco biomarkers in neonatal blood (which reflects perinatal exposure) and found positive associations with retinoblastoma [73]. Regarding radon-222, the advisory group noted an increased number of studies from the latest evaluation [13] on childhood leukaemia which warrant a systematic evaluation through a Monographs evaluation [53]. Several metrics of outdoor air pollution (e.g., traffic density, PM2.5) have been associated with childhood leukaemia, including in a pooled analysis of traffic density exposure [relative risk (RR) 1.09; 95% confidence interval (CI): 1.00–1.20 for overall childhood leukaemia) and benzene exposure, especially for AML (meta-RR 1.84; 95% CI: 1.31–2.59) [40] or other subtypes of childhood cancer [41].

### Cannabis smoking

In addition to tobacco smoking, cannabis smoking, which has never been evaluated by the IARC Monographs, was given high priority for evaluation by the IARC Advisory Group, mainly for emerging evidence related to testicular cancer, which is common in adolescents and young adults. The IARC Monographs meeting for evaluating cannabis smoking is scheduled for 3–10 November 2026. There are sporadic positive findings associating parental cannabis smoking with subtypes of childhood cancers (childhood leukaemia, astrocytoma, rhabdomyosarcoma and neuroblastoma) in case–control studies conducted in the 1990s and early 2000s, mostly by the Children’s Cancer Study Group [31]. However, these findings are limited to single studies. Since then, no further studies on childhood cancer and parental cannabis smoking were identified. A 2009 carcinogenicity evaluation of cannabis smoke by the state of California considered that there was suggestive evidence of increased cancer risk in the offspring of parents who smoked marijuana, based on these studies [32]. In 2017, a comprehensive review on cancer risk by the US National Academy of Science highlighted the need for research on parental cannabis smoking and cancer in offspring [74]; however, we identified no recent studies on this topic.

### Component of air pollution

In addition to outdoor air pollution in its entirety, several components of air pollution (e.g., ultrafine particles, nitrogen dioxide (NO_2_), ozone, acetaldehyde, carbon tetrachloride, selenium) have been recommended for evaluation by IARC Monographs (Table 1) [53]. Some of these components were studied in relation to childhood cancer risk in studies that coupled geocoded residential information with air pollution modelling data. Overall, the associations reported are limited to single findings in single studies. Ultrafine particles were found to be associated with increased risk of childhood cancer overall, but not specific subtypes, in a case–control study based on record linkage data in Ontario [42]. A previous study from the same dataset found an association between NO_2_ and childhood ALL [48]. Acetaldehyde in air pollution in the first year of life was associated with retinoblastoma [43] and, in the third trimester, with Wilms tumour [44]. Other outdoor air pollution components recommended with a high (formaldehyde, carbon tetrachloride) or medium (xylenes, selenium) priority have also been found to be sporadically associated with Wilms tumour [44], childhood neuroblastoma [51], and germ cell tumour [49] or childhood leukaemia, respectively (Table 1).

Thus far, the air pollution components prioritised for evaluation by the IARC Monographs have been limited to sporadic findings in few datasets mainly based in North America. Therefore, especially for the rarer childhood cancer subtypes, positive results would need to be confirmed in other populations to strengthen causal inference for future evaluation.

### Pesticides

Several pesticides have been prioritised by the IARC Advisory Group for Monographs evaluation. Exposure of children to pesticides can occur through residential use, contamination of the environment from nearby agricultural applications or from parental occupational exposure [70]. Early observations that exposure to pesticides in general could increase the risk of several types of childhood cancers relied mainly on case–control studies using parental reporting of personal or occupational use of pesticides, without specification of pesticide names [75,76]. This type of evidence has also increased for solid childhood cancer subtypes [30]; however, such generic classification of exposure is not generally informative for a Monographs evaluation, unless it is possible to infer an exposure to a specific pesticide. No individual pesticide has as yet been listed as a specific cause of childhood cancer by the IARC Monographs, owing to the paucity of data on individual pesticides and specific childhood cancers. Most existing cohort studies of the children of farmers and pesticide applicators have had few cancer endpoints or have been unable to identify specific pesticides of concern [77–79].

To date, the few childhood cancer studies with specific pesticide exposures are all case–control studies [53,80]. In addition to parental reports on types of pesticides used [58], novel methods are beginning to emerge using geocoded residential history [59], or using home dust measurements [57]. While house dust is taken after diagnosis, given the long half-life of some pesticides in household dust, it can be considered long-term exposure among those with stable residences [57]. However, exposure misclassification with this type of measurement cannot be excluded, especially for less persistent pesticides [81].

Few studies with pesticide-specific data were identified by the IARC Advisory Group when prioritising pesticides for evaluation (Table 1). Using geocoding-based exposure assessment, a state-wide record linkage case–control study in California reported that permethrin and pendimethalin (recommended for evaluation with high and medium priority, respectively) were associated with childhood leukaemia (<16 years of age) [54]. In another California geocoded state-wide case–control study, pendimethalin, chlorpyrifos and malathion (recommended with high priority) were associated with childhood ALL (<6 years old); however, models did not account for exposure to other pesticides [59]. Positive results were also reported for maternal self-reported permethrin exposure (at home or in the workplace) and childhood AML and ALL in Brazil [58]. However, in the California Childhood Leukemia Study, which measured exposure in household dust, permethrin and chlorpyrifos were not associated with childhood ALL [57].

### Polyfluoroalkyl substance (PFAS)

Perfluorohexane sulfonic acid (PFHxS), a PFAS, was recommended for IARC Monographs evaluation with high priority, based on mechanistic evidence [53]. A recent study, using a nested case–control design within a Finnish maternal cohort, used maternal sera taken during pregnancy to assess in utero PFAS exposure and the subsequent risk of childhood ALL. In this study, PFHxS was measured, but not found to be associated with the risk of childhood ALL; however, another PFAS, N-methylperfluorooctane sulfonamidoacetic acid (MeFOSAA), was positively associated with childhood ALL [82]. Likewise, a case–control study from the Childhood Record Linkage Study in California found that MeFOSAA concentrations, measured in neonatal stored blood samples, were associated with childhood ALL, particularly in children younger than two years of age [83]. At the time of the PFOA and PFOS IARC evaluation, only one study that used neonatal bloodspots was available, a retinoblastoma case–control study based on California registry data [84].

### Other agents prioritised

Parental occupations have been also studied in relation to childhood cancer in offspring, and the IARC Monographs identified maternal occupation as a painter as a possible cause of childhood leukaemia [18] (Fig. 1). Textile manufacturing work, currently classified in Group 2B, has been assigned a high priority for re-evaluation given new epidemiological evidence for nasal, lung and urinary bladder cancer. In addition, several studies associated paternal textile industry work with increases of several type of childhood cancer in offspring [33,34,36].

Regarding prioritised biological agents with childhood cancer evidence, in addition to HCMV, which was given a high priority in the 2024 Advisory Group review and was soon after evaluated [11], the IARC Advisory Group also recommended *Toxoplasma gondii* with medium priority. They noted a positive association between positive serology for *T. gondii* and childhood brain tumours or childhood leukaemia based on a single study with post-diagnostic samples [70]. No studies using pre-diagnostic or nested case–control studies were identified.

## Conclusions

While few modifiable risk factors have been established as causes of childhood cancer, two IARC Monographs evaluations in 2025 established that there is *limited* evidence that automotive gasoline and HCMV are causes of childhood ALL. This highlights a potentially larger role for environmental factors in the aetiology of childhood cancer than previously appreciated and the need for additional epidemiological data informative for cancer hazard identification.

The recent classifications of automotive gasoline and HCMV reflect advancements in childhood cancer aetiological epidemiological research, particularly in studies that are able to obtain pre-diagnostic exposure assessment data through the exploitation of historical residential location data or stored pre-diagnostic biological samples, including neonatal bloodspots.

Among the agents prioritised for evaluation by the IARC Monographs programme, with evidence of increased risks for some types of childhood cancers, the data are currently generally restricted to single studies with positive findings.

In conclusion, aetiological factors for childhood cancer are increasingly being identified, and epidemiological research in this area has evolved to incorporate novel approaches for pre-diagnostic exposure assessment. These approaches move away from relying solely on interview-based information or biological samples collected at diagnosis, which are subject to recall bias and reverse causality, respectively. Future Monographs evaluations are likely to be informed by studies with pre-diagnostic exposure assessments. Studies conducted in different geographical locations, focusing on specific cancer types, including leukaemia subtypes, and relevant exposure windows, seem likely to be informative.

## Data Availability

Data were extracted from the IARC Monographs websites. The list of classifications, including by cancer type, is continuously updated, maintained and freely available at https://monographs.iarc.who.int/agents-classified-by-the-iarc/.
